# Hybrid Mice Reveal Parent-of-Origin and *Cis*- and *Trans*-Regulatory Effects in the Retina

**DOI:** 10.1371/journal.pone.0109382

**Published:** 2014-10-23

**Authors:** Susan Q. Shen, Ernest Turro, Joseph C. Corbo

**Affiliations:** 1 Department of Pathology and Immunology, Washington University School of Medicine, St. Louis, Missouri, United States of America; 2 Cancer Research UK Cambridge Institute, University of Cambridge, Cambridge, United Kingdom; 3 Department of Haematology, University of Cambridge, National Health Service Blood and Transplant, Cambridge, United Kingdom; University of Cologne, Germany

## Abstract

A fundamental challenge in genomics is to map DNA sequence variants onto changes in gene expression. Gene expression is regulated by *cis*-regulatory elements (CREs, i.e., enhancers, promoters, and silencers) and the *trans* factors (e.g., transcription factors) that act upon them. A powerful approach to dissecting *cis* and *trans* effects is to compare F1 hybrids with F0 homozygotes. Using this approach and taking advantage of the high frequency of polymorphisms in wild-derived inbred Cast/EiJ mice relative to the reference strain C57BL/6J, we conducted allele-specific mRNA-seq analysis in the adult mouse retina, a disease-relevant neural tissue. We found that *cis* effects account for the bulk of gene regulatory divergence in the retina. Many CREs contained functional (i.e., activating or silencing) *cis*-regulatory variants mapping onto altered expression of genes, including genes associated with retinal disease. By comparing our retinal data with previously published liver data, we found that most of the *cis* effects identified were tissue-specific. Lastly, by comparing reciprocal F1 hybrids, we identified evidence of imprinting in the retina for the first time. Our study provides a framework and resource for mapping *cis*-regulatory variants onto changes in gene expression, and underscores the importance of studying *cis*-regulatory variants in the context of retinal disease.

## Introduction

Photoreceptors mediate vision by converting light into an electrical signal, which is then processed by the inner retina and sent to the brain as visual information. Photoreceptors constitute the vast majority (>70%) of cells in the mouse retina [1], and they are prominent targets for disease: the majority of more than 200 genetic forms of retinal degeneration affect photoreceptors [2]. Many of the key transcriptional regulators in photoreceptor development are known, and the transcriptomes of these cells have been profiled over normal development as well as in disease states [3]–[5]. Furthermore, the regulatory regions of mature photoreceptors in adult mouse retinas have been mapped genome-wide, based on the binding patterns of two key photoreceptor transcription factors, CRX (cone-rod homeobox) [6] and NRL (neural retina leucine zipper) [7], as well as the patterns of ENCODE DNaseI hypersensitivity sequencing (DNase-seq) data [8]. Photoreceptors therefore represent a disease-relevant cell type well-suited for studying the mechanisms of mammalian gene regulation.

Changes in gene expression give rise to cell-type identity, intraspecies variation, and interspecies diversity, thereby acting as the molecular underpinnings for development, disease, and evolution, respectively [9], [10]. Alterations in gene expression can arise from changes in *cis*-regulatory elements (CREs, i.e., enhancers, promoters, and silencers), or from changes in the *trans* factors (e.g., transcription factors) that interact with CREs. To distinguish between *cis* and *trans* effects, a powerful approach is to compare F1 heterozygous hybrids with F0 homozygotes. In F1 hybrids, both alleles of a gene are contained within the same nucleus and are exposed to the same set of *trans* factors. A *trans*-regulatory difference (“*trans* effect”) manifests as conserved expression between the two alleles in the F1 hybrids, despite differential expression of the gene in the F0 homozygotes. In contrast, a *cis*-regulatory difference (“*cis* effect”) manifests as an allelic expression imbalance (AEI)—i.e., differential expression between the two alleles of a gene in the F1 hybrids, with an allelic ratio that recapitulates the ratio of gene expression levels in the F0 homozygotes. By measuring allele-specific gene expression, the relative contributions of *cis* and *trans* effects can be dissected genome-wide. AEI can also arise from parent-of-origin effects (e.g., imprinting). Importantly, by conducting reciprocal crosses, parent-of-origin effects can be identified and filtered to avoid confounding the analysis of *cis* and *trans* effects.

Prior studies utilizing the F1 hybrid study design in yeast and *Drosophila* have yielded a range of results: earlier pyrosequencing and microarray-based studies found that *cis* effects predominate [11], [12], while more recent RNA-seq studies indicate a greater role for *trans* effects [13], [14]. Regardless, all studies acknowledge a high prevalence of *cis* effects. The F1 hybrid study design has been used to investigate gene regulation in one mammalian tissue thus far, the mouse liver [15]. In that study, the authors found that *cis* and *trans* effects often act together in opposite directions, with the net effect of stabilizing gene expression. Here, we conduct an F1 hybrid study using allele-specific mRNA-seq analysis to chart the regulatory landscape of a portion of the mature mammalian central nervous system, the adult mouse retina. We utilize two distantly related strains of mice, Cast/EiJ and C57BL/6J, whose retinas are known to exhibit phenotypic differences [16], [17]. The primary goal of our study is to dissect the contributions of *cis* and *trans* effects on gene regulation in photoreceptors. As part of our study, we identify parent-of-origin effects in the retina, a tissue in which imprinting has not previously been studied. By re-analyzing available liver data [15] and comparing them to our data from the retina, we assess the degree of tissue specificity of the observed *cis*- and *trans*-regulatory effects. Furthermore, we integrate our gene expression data with knowledge about the location of CREs, thereby providing insights into the effects of *cis*-regulatory variants on gene expression.

## Results

**Figure 1:**
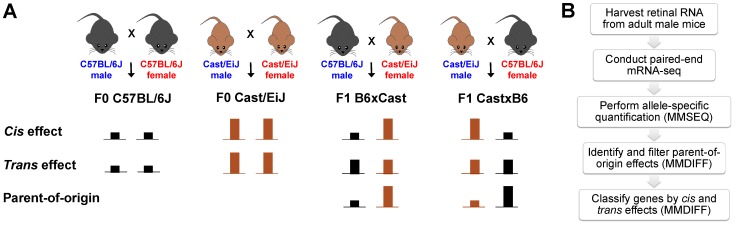
Study design. (A) F0 and F1 mice were generated via the depicted crosses. The schematic diagram illustrates example expression patterns for a *cis* effect, *trans* effect, and parent-of-origin effect. For a *cis* effect, in the F1 hybrids, the Cast/EiJ allelic expression relative to the C57BL/6J allelic expression recapitulates the ratio of gene expression levels in the F0 homozygotes. For a *trans* effect, the F1 hybrids express the Cast/EiJ and C57BL/6J alleles equally. For a parent-of-origin effect, there is preferential expression of the maternal allele (as depicted) or the paternal allele, as seen by comparison of the reciprocal F1 hybrids. (B) An overview of the workflow is shown.

The ancestors of two inbred *Mus musculus* strains, the standard reference strain C57BL/6J and the wild-derived inbred strain Cast/EiJ, diverged ∼1 million years ago [18]. Cast/EiJ harbors ∼18 million single nucleotide polymorphisms (SNPs) and ∼3 million insertions/deletions (indels) relative to C57BL/6J, involving nearly 1% of the accessible genome [19]. In addition, Cast/EiJ retinas show substantial phenotypic differences, namely reduced photopic and scotopic electroretinogram amplitudes compared to C57BL/6J retinas [16], [17]. We reciprocally crossed these two strains to obtain four genotypic classes for analysis (Figure 1A): F0 C57BL/6J, F0 Cast/EiJ, F1 B6xCast (resulting from C57BL/6J male×Cast/EiJ female), and F1 CastxB6 (resulting from Cast/EiJ male×C57BL/6J female). For each class, we analyzed three biological replicates, each consisting of a pool of retinas.

We collected retinas from adult mice at age 8 weeks, a time point at which mouse retinal CRX ChIP-seq [6] and ENCODE DNase-seq [8] were previously conducted. To control for sex-linked effects and because the X chromosome of Cast/EiJ is preferentially expressed in F1 hybrid females [20], we used retinas from male mice only and focused our analyses on autosomal genes. We conducted paired-end mRNA-seq and calculated gene expression for F0 samples and allele-specific expression for F1 samples by mapping reads to the C57BL/6J and Cast/EiJ transcriptomes using MMSEQ (Figure 1B; see Methods) [21].

We verified that biological replicates for each F0 or F1 class exhibited a high degree of agreement for gene expression or allele-specific expression estimates, respectively (Table 1 and Table 2). We also verified the accuracy of our mapping strategy by examining the X chromosomal reads in the F1 samples. Since samples derived solely from male retinas, the X chromosomal reads should map exclusively to the maternal genome. Accordingly, X chromosomal reads for F1 B6xCast should map to Cast/EiJ, while those for F1 CastxB6 should map to C57BL/6J. In validation of our mapping strategy, we found high accuracy (>99%) of X chromosomal reads for all F1 samples (Table 3). Importantly, the accuracy of mapping to the X chromosome of F1 B6xCast and F1 CastxB6 samples was similar, indicating that there was no substantial read-mapping bias toward the standard reference genome, C57BL/6J, a potential confounding factor in the allele-specific quantification [22].

**Table 1:** Agreement between F0 biological replicates.

		F0 C57BL/6J			F0 Cast/EiJ		
		R1	R2	R3	R1	R2	R3
F0 C57BL/6J	R1	1					
	R2	**0.983**	1				
	R3	**0.992**	**0.982**	1			
F0 Cast/EiJ	R1	0.873	0.814	0.876	1		
	R2	0.912	0.893	0.919	**0.956**	1	
	R3	0.907	0.912	0.904	**0.897**	**0.979**	1

Pearson *r* values for FPKM (fragments per kb of transcripts per million mapped read pairs) estimates across F0 samples. Bold denotes comparison between biological replicates (R1, R2, and R3) of the same genotype.

**Table 2:** Agreement between F1 biological replicates.

		F1 B6xCast, B6 allele			F1 B6xCast, Cast allele			F1 CastxB6, B6 allele			F1 CastxB6, Cast allele		
		R1	R2	R3	R1	R2	R3	R1	R2	R3	R1	R2	R3
F1 B6xCast, B6 allele	R1	1											
	R2	**0.929**	1										
	R3	**0.975**	**0.940**	1									
F1 B6xCast, Cast allele	R1	0.949	0.911	0.953	1								
	R2	0.937	0.922	0.946	**0.973**	1							
	R3	0.923	0.918	0.941	**0.977**	**0.960**	1						
F1 CastxB6, B6 allele	R1	0.932	0.971	0.960	0.914	0.924	0.926	1					
	R2	0.927	0.974	0.952	0.911	0.920	0.916	**0.987**	1				
	R3	0.939	0.957	0.965	0.925	0.924	0.935	**0.985**	**0.975**	1			
F1 CastxB6, Cast allele	R1	0.885	0.947	0.923	0.926	0.943	0.939	0.951	0.958	0.950	1		
	R2	0.909	0.909	0.945	0.942	0.940	0.961	0.948	0.928	0.963	**0.965**	1	
	R3	0.906	0.917	0.947	0.946	0.938	0.959	0.951	0.947	0.948	**0.971**	**0.979**	1

Pearson *r* values for FPKM estimates across F1 samples. Bold denotes comparison between biological replicates (R1, R2, and R3) of the same genotype and for the same allele (B6 allele or Cast allele).

**Table 3:** Accuracy of X chromosomal read mapping in F1 samples.

F1 B6xCast		F1 CastxB6	
Maternal allele: Cast/EiJ		Maternal allele: C57BL/6J	
R1	99.4%	R1	99.5%
R2	99.5%	R2	99.5%
R3	99.5%	R3	99.5%

Percentages of X chromosomal unique hits (i.e., read pairs mapping uniquely to C57BL/6J or Cast/EiJ) that mapped to the correct genome. Since only males were used, reads should derive only from the maternal allele.

### Strongly imprinted genes in other tissues show evidence of imprinting in the retina

To evaluate *cis* and *trans* effects on gene expression in the retina, we first needed to filter genes affected by parent-of-origin effects (e.g., imprinting). Genomic imprinting is an epigenetic phenomenon that causes an imbalance in allelic expression depending on whether the allele is maternally or paternally derived [23]. In the extreme case, one allele is completely silenced, rendering the locus functionally monoallelic; for this reason, many mutations in imprinted loci are associated with human disease [24]. Differential methylation of alleles provides a molecular basis for imprinting, but because methylation can occur in a tissue-specific manner, a gene can be imprinted in one tissue but not another, despite being expressed in both [25]. Although imprinting has been extensively studied in a number of human and mouse tissues, including brain and placenta [25]–[27], it has not previously been studied in the retina.

**Figure 2:**
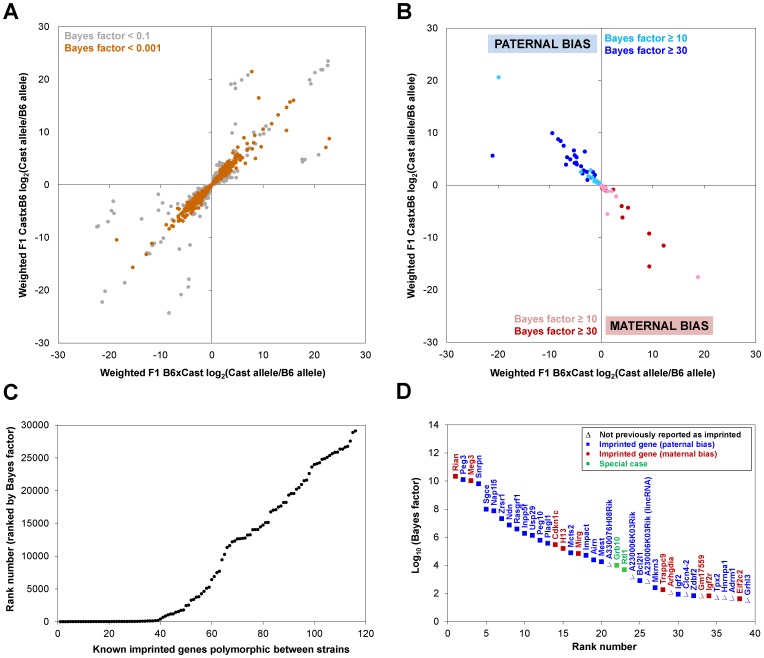
Characterization of parent-of-origin effects in the retina. Autosomal genes polymorphic between C57BL/6J and Cast/EiJ were analyzed in retinas from reciprocal F1 hybrids. Higher Bayes factors indicate greater likelihood of imprinting. (A) Non-imprinted genes with Bayes factor <0.1 (gray) and <0.001 (orange) are depicted. (B) Parent-of-origin effects with preferential expression of the paternal (blue) or maternal (red) allele with Bayes factor ≥10 (light) and ≥30 (dark) are depicted. (C) Top-ranked (low rank number) genes are enriched for known imprinted genes. (D) Genes with strong evidence of imprinting in the retina (Bayes factor ≥30) that exhibit preferential expression of the paternal (blue) or maternal (red) allele are depicted. Green, special cases—see text for discussion of *Rtl1* and *Grb10*. Filled squares, genes previously reported as imprinted in other tissues. Empty triangles, not previously reported as imprinted. A230006K03Rik appears twice because it is associated with two Ensembl ID's, a protein-coding gene and a lincRNA.

By analyzing the reciprocal F1 hybrids, we identified autosomal genes that exhibited a significant maternal bias (maternally expressed, paternally silenced) or paternal bias (paternally expressed, maternally silenced) (Figure 2A and 2B; Supporting Information S1). To determine whether these genes have been identified as imprinted in other tissues, we searched for known imprinted mouse genes in four databases (see Methods). Using a Bayesian model selection approach implemented in the MMDIFF program [28], we ranked genes in our dataset by the probability of imprinting and observed a clear enrichment of known imprinted genes among highly-ranked genes (Figure 2C). Among the top-ranked genes, the vast majority were well-characterized imprinted genes listed in multiple imprinting databases (see Methods) and displayed the same allelic bias as previously reported (Figure 2D).

We identified 75 genes as highly likely to be imprinted (Bayes factor ≥10). Among these, 39 genes were extremely likely to be imprinted (Bayes factor ≥30), of which 29/39 (74%) were known imprinted genes. In 27 out of 29 cases, the direction of parental bias that we observed was consistent with that reported in the literature. For instance, *Peg3* (paternally expressed 3) and *Meg3* (maternally expressed 3) were our 2^nd^ and 3^rd^ ranked imprinting genes, respectively. *Igf2* and *Igf2r* were our 30^th^ and 34^th^ ranked imprinted genes, respectively. *Igf2* and its receptor *Igf2r* were the first imprinted mouse genes discovered and remain among the best-characterized, with paternally expressed *Igf2* promoting growth and maternally expressed *Igf2r* inhibiting growth [29], [30]. Consistent with an emerging view of imprinting occurring on a spectrum rather than being an all-or-none event [15], [27], we found varying degrees of allelic bias even for well-characterized imprinted genes, ranging from subtle (e.g., <2-fold preference for the maternal over the paternal allele of *Igf2r*) to extreme (e.g., >1000-fold preference for the maternal over the paternal allele of *Rian*).

*Rtl1*, also known as *Peg11*, is a gene in the *Dlk1-Dio3* imprinted cluster [31]. In our dataset, reads mapped preferentially to the maternal allele at the *Rtl1* locus. Previous studies in other tissues found that *Rtl1* is expressed from the paternal allele, while an antisense RNA, *anti-Rtl1*, is transcribed from the same locus on the maternal allele and gives rise to two maternally expressed microRNAs [31], [32]. Since our RNA-seq was not strand-specific, we could not discern whether *Rtl1* or *anti-Rtl1* is maternally expressed in the adult mouse retina.

*Grb10* is unique among imprinted genes in that it exhibits opposite patterns of imprinting depending on the tissue where it is expressed. In adult mice, *Grb10* is maternally expressed in some tissues, such as muscle and adipose, where it plays a role in glucose metabolism [33]. However, it is paternally expressed in the brain, where it affects social behavior [34]. This tissue-specific parent-of-origin effect is associated with usage of a paternal-specific *Grb10* promoter during neural fate commitment [35]. Interestingly, in the retina, we found that *Grb10* follows the pattern of muscle and adipose tissue, with preferential expression of the maternal allele. Thus, although the retina belongs to the central nervous system, it does not follow the imprinting pattern observed in the brain for this locus.

Together, these analyses indicate that imprinting occurs in the retina, and that the pattern of imprinting is largely, but not always, concordant between the retina and the brain. Notably, the developing retina expresses the DNA methyltransferase DNMT3A, which is required for the germline methylation of imprinted loci [36], [37]. Methylation analysis (e.g., bisulfite sequencing) of the retina would confirm whether the parent-of-origin effects identified here correspond to differentially methylated regions (DMRs), as methylation-independent parent-of-origin effects have also been reported [26], [38].

### One-third of differentially expressed genes between Cast/EiJ and C57BL/6J retinas are associated with photoreceptor CREs

Previous microarray studies have suggested substantial gene expression differences between C57BL/6J and Cast/EiJ retinas [17]. Thus, we surveyed differentially expressed (DE) genes between the adult male F0 Cast/EiJ and F0 C57BL/6J retinas. We identified 3,799 autosomal DE genes between the F0 samples at a false discovery rate (FDR) of 5% using DESeq [39] (Supporting Information S2). Among these, 1,701/3,799 (45%) showed higher expression in Cast/EiJ.

CRX is a key photoreceptor transcription factor required for the expression of many rod and cone genes [40], [41]. Previous CRX ChIP-seq studies conducted in adult C57BL/6 mouse retinas demonstrated that CRX-bound regions (CBRs) demarcate both known and putative photoreceptor CREs [6]. CBRs have a propensity to cluster around genes expressed in photoreceptors, and knowledge of CBR locations has helped pinpoint novel human retinal disease genes [42], [43].

We used available adult mouse retinal CRX ChIP-seq data to determine whether the differentially expressed genes were CBR-associated [6]. We found that among all 34,964 autosomal genes, 6,257 (18%) had at least one CBR assigned to them. However, among the 3,799 DE genes between the two strains, 1,275 (34%) were CBR-associated, representing a significant enrichment (P<10^−14^, hypergeometric distribution). Thus, among all autosomal genes, those that were differentially expressed between Cast/EiJ and C57BL/6J were more likely to be CBR-associated.

**Figure 3:**
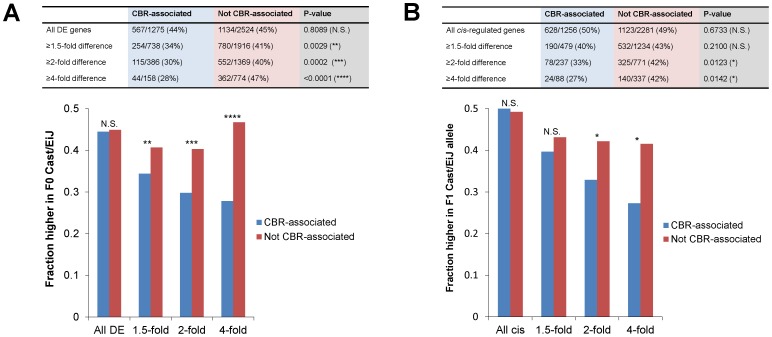
Comparison of differentially expressed and *cis*-effect genes associated with photoreceptor CREs. Genes were classified as being associated with CRX ChIP-seq peaks (CBR-associated) or not. (A) Differentially expressed (DE) autosomal genes were identified using DESeq at 5% FDR. The proportions of genes with higher expression in F0 Cast/EiJ than F0 C57BL/6J at various fold changes are shown. (B) *Cis*-effect autosomal genes were identified using MMDIFF. Proportions of genes with higher expression in F1 Cast/EiJ allele than F1 C57BL/6J allele at various fold changes are shown. P-values were calculated with two-tailed Fisher's exact test. N.S. = not significant, *<0.05, **<0.01, ***<0.001, **** <0.0001.

Furthermore, differentially expressed CBR-associated genes more often had lower expression in Cast/EiJ than C57BL/6J when compared to differentially expressed non-CBR-associated genes (Figure 3A). This effect was especially pronounced for genes with greater fold change between the two strains. Together, these findings suggest that Cast/EiJ overall has lower expression of photoreceptor genes than C57BL/6J, consistent with a previous microarray analysis [17]. The physiological function of rods, which constitute >97% of the photoreceptors in the mouse retina [44], can be measured by the a-wave of the scotopic electroretinogram (ERG). Interestingly, the gene expression differences may be reflected in the rod photoreceptor physiology of Cast/EiJ, which has a scotopic a-wave amplitude ∼40–50% that of C57BL/6J, despite intact morphology [17], [45].

### *Cis*-regulatory effects account for the bulk of gene regulatory divergence between Cast/EiJ and C57BL/6J retinas

**Figure 4:**
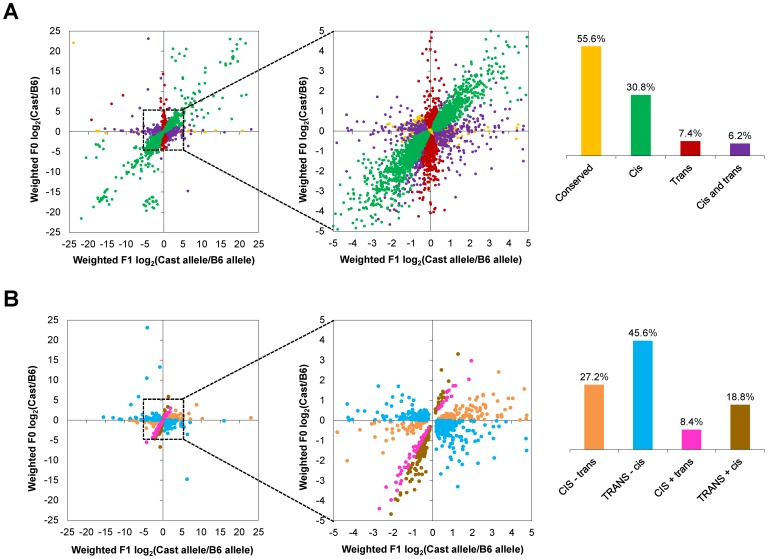
Classification of genes by mechanism of gene regulatory divergence. (A) Genes were classified as conserved (yellow; largely obscured), *cis* (green), *trans* (red), or *cis* and *trans* (purple). (B) *Cis*- and *trans*-effect genes were further subcategorized as to whether the *cis* and *trans* effects acted in the same (“+” sign; pink and brown) or opposite (“−” sign; orange and blue) directions, and whether the *cis* (CAPS; orange and pink) or *trans* (CAPS; blue and brown) effect was stronger. Insets, magnified views.

Next, we determined whether gene expression divergence was attributable to *cis* effects, *trans* effects, or a combination of both. For this analysis, we examined allele-specific expression in the F1 hybrids in conjunction with gene expression in the F0 parents (Figure 4A; Supporting Information S3). After excluding 306 genes with an imprinting Bayes factor >3, we were able to classify 11,484 autosomal genes with high confidence (see Methods). Among these, 6,380/11,484 (56%) were best modelled as conserved (i.e., no significant difference), 3,537/11,484 (31%) as divergent due to *cis* effects, 850/11,484 (7%) as divergent due to *trans* effects, and 717/11,484 (6%) as divergent due to a combination of *cis* and *trans* effects. Therefore, *cis*-regulatory effects were the primary mechanism of gene regulatory divergence between Cast/EiJ and C57BL/6J retinas.

We then subcategorized the genes whose divergence was due to a combination of *cis*- and *trans* effects into the following classes: (1) *CIS*−*trans* (*cis* and *trans* effects acting in opposite directions, with *cis* effects stronger) had 195/717 (27%) of the genes, (2) *TRANS−cis* (*cis* and *trans* effects acting in opposite directions, with *trans* effects stronger) had 327/717 (46%) of the genes, (3) *CIS+trans* (*cis* and *trans* effects acting in the same direction, with *cis* effects stronger) had 60/717 (8%) of the genes, and (4) *TRANS+*cis (*cis* and *trans* effects acting in the same direction, with *trans* effects stronger) had 135/717 (19%) of the genes (Figure 4B).

When *cis* and *trans* effects acted together in the retina, they acted in opposite directions to stabilize gene expression in the majority (522/717 or 73%) of cases, while they acted in the same direction to shift gene expression in a minority (195/717 or 27%) of cases. However, the primary mechanism of gene regulatory divergence was *cis*-regulatory effects acting with little or no contribution from *trans*-regulatory effects, accounting for 3,537/5,104 (69%) of gene regulatory divergence. This suggests that functional *cis*-acting sequence variants in the Cast/EiJ genome often drive altered gene expression.

We further examined the 3,537 *cis*-effect genes, of which 1,751/3,537 (50%) showed higher expression of the Cast/EiJ allele than the C57BL/6J allele, and of which 1,256/3,537 (36%) were CBR-associated. We found that *cis*-effect genes that were CBR-associated more often had lower Cast/EiJ allele expression than *cis*-effect genes that were not CBR-associated, for genes with higher fold change between the two alleles (Figure 3B). These results are consistent with the notion that the Cast/EiJ genome overall harbors many *cis*-regulatory variants whose net effect is to diminish photoreceptor gene expression.

*Trans* effects could arise from differential activity of transcription factors. Therefore, we inspected the rod photoreceptor transcriptional network, whose members include the transcription factors CRX, RORβ, NRL, and NR2E3 [5]. We found that *Crx* and *Nrl* transcript levels were both conserved in the F0 and F1 retinas. *Rorb* was a solely *trans*-effect gene, with lower expression in Cast/EiJ, suggesting that the upstream regulators of RORβ in the retina (whose identities are unknown) have altered activity in Cast/EiJ. *Nr2e3* was also a solely *trans*-effect gene, with higher expression in Cast/EiJ. Since NR2E3 is known to be regulated by CRX and NRL [46], and the mRNA levels of *Crx* and *Nrl* were unaltered, we examined whether CRX or NRL harbored coding mutations that might alter their protein activity. However, we did not find any non-synonymous mutations in *Nrl* or in the best-characterized isoform of *Crx* [41], [47]. Thus, we identified differential *trans*-regulation of *Rorb* and *Nr2e3* in Cast/EiJ relative to C57BL/6J, but the reasons for these *trans* effects are unknown.

### Higher frequency of variants in photoreceptor CREs correlates with differential expression

**Figure 5:**
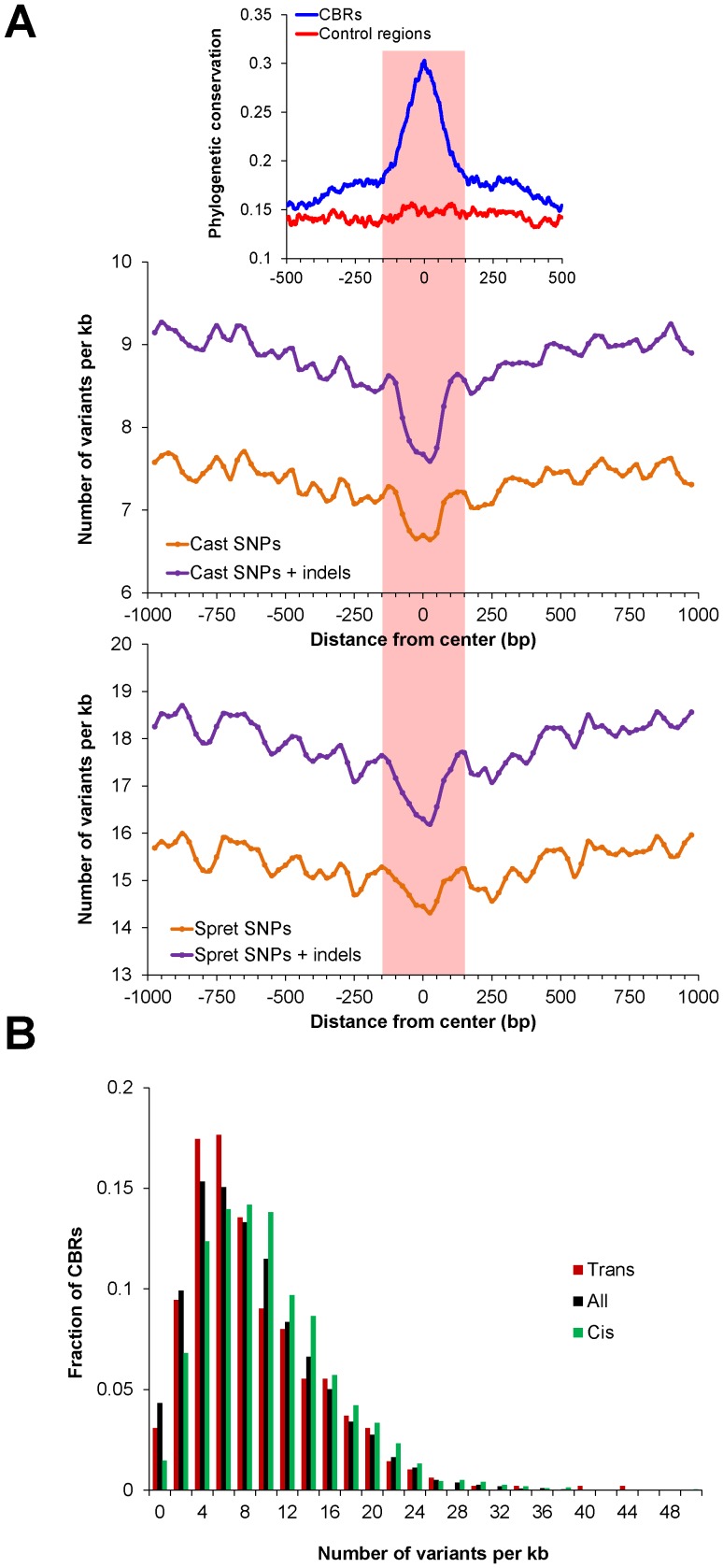
Analysis of variant density in photoreceptor CREs. (A) The number of Cast/EiJ (top) or Spret/EiJ (bottom) SNPs and indels relative to C57BL/6J was determined in 50 bp windows (sliding 25 bp at a time) across the 2 kb region centered on CBRs. Phylogenetic conservation for CBRs is based on PhastCons scores as found in [6]. The highlighted area corresponds to the central 300 bp region. (B) Histogram showing frequency of variants (SNPs+indels) in the 1 kb region centered on all CBRs (black), CBRs associated with *cis*-effect genes (green), and CBRs associated with *trans*-effect genes (red). Total bar height was normalized to 1 for each category.

Whereas *trans*-regulatory effects are due to the levels or activities of upstream signaling cascades and transcriptional regulators (e.g., transcription factors), *cis*-regulatory effects can arise from functional *cis*-acting variants within CREs. We undertook a survey of Cast/EiJ variants relative to C57BL/6J that fell within CBRs. First, we asked whether CBRs were depleted or enriched for Cast/EiJ variants by tabulating the number of SNPs and indels across the 2 kb region centered on CBRs. We found that the frequency of variants decreased toward the center of CBRs (Figure 5A). The bulk of the depletion occurred within the central 300 bp, consistent with the previous finding that phylogenetic conservation, as measured by PhastCons scores [48], is markedly elevated within the central region of CBRs [6]. Also consistent with this result, a recent functional analysis of ∼1,300 CBRs in the mouse retina demonstrated that short fragments corresponding to the central 84 bp of CBRs possess substantial *cis*-regulatory activity [49]. When we conducted the same analysis of variant depletion for Spret/EiJ, an inbred strain of *Mus spretus* that diverged from *Mus musculus* ∼2 million years ago [50], we obtained similar results (Figure 5A). Thus, CBRs are functionally constrained and have likely undergone selection in the mouse lineage, particularly in the central-most portion of the CBR.

If *cis* effects are due to altered transcriptional activity driven by *cis*-regulatory variants, we would expect to find a higher frequency of functional variants in the CREs around *cis*-effect genes compared to the *trans*-effect genes. We first observed that the proportion of genes that were CBR-associated was approximately equal across categories: 2,149/6,380 (34%) of conserved genes, 1,256/3,537 (36%) of *cis*-effect genes, 300/850 (35%) of *trans*-effect genes, and 242/717 (34%) of *cis*- and *trans*-effect genes. We then tabulated the Cast/EiJ variants (SNPs and indels) within the central 1 kb centered on the CBRs associated with each gene (Supporting Information S4). For all 10,212 CBRs, we found 86,389 variants, for a frequency of 8.46 variants per kb. When we examined the *cis*-effect genes, we found 21,174 variants in the 2,185 associated CBRs, for a frequency of 9.69 variants per kb. This was significantly higher than the variant frequency in all CBRs (P<10^−14^, hypergeometric distribution). In contrast, for the *trans*-effect genes, we found 4,068 variants in 487 CBRs, corresponding to a frequency of 8.35 variants per kb, which was not significantly different from the variant frequency in all CBRs (P = 0.2, hypergeometric distribution). The tendency for CBRs associated with *cis*-effect genes to have a higher frequency of variants than CBRs associated with *trans*-effect genes is also evident from the distributions of variants across individual CBRs (Figure 5B). Collectively, we find that CBRs associated with *cis*-effect genes are enriched for variants, whereas CBRs associated with *trans*-effect genes are not. These results suggest that CBRs contain functional *cis*-regulatory variants that alter transcriptional activity, but future empirical testing is needed to demonstrate the causality of specific variants.

### The Cast/EiJ genome harbors both activating and silencing *cis*-regulatory variants associated with retinal disease genes

Given that hundreds of genes can contribute to retinal disease, we asked whether any of the *cis*-effect genes were associated with human retinopathies (Supporting Information S5). We found 62 *cis*-effect genes with human orthologues that were listed in the RetNet database, an up-to-date and comprehensive compendium of retinal disease genes [51]. Of these, 30/62 (48%) showed higher expression of the Cast/EiJ allele. Therefore, although Cast/EiJ mice have diminished rod and cone ERG responses compared to C57BL/6J, they harbor both activating and silencing *cis*-regulatory variants.

**Figure 6:**
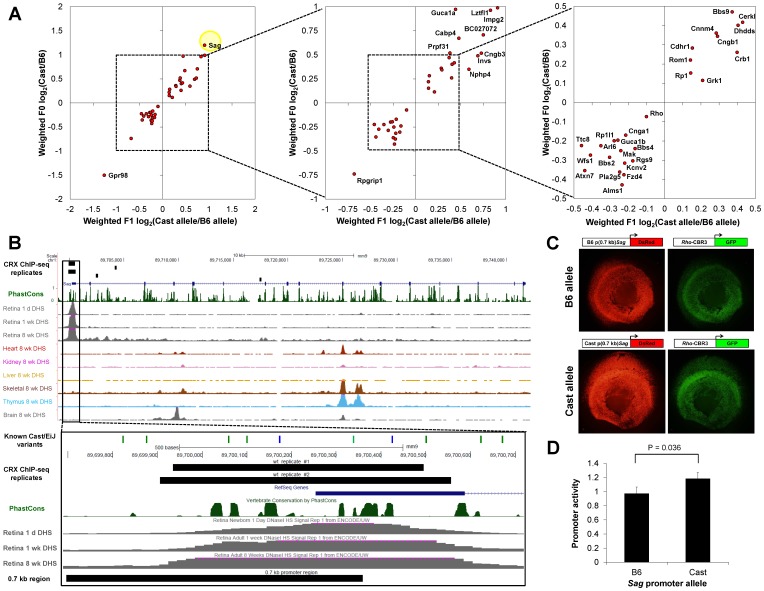
*Cis*-effect genes associated with retinal disease and photoreceptor CREs. (A) *Cis*-effect genes associated with CRX ChIP-seq peaks were matched against the RetNet database of retinal disease genes. The yellow circle highlights *Sag*. (B) *Sag* locus, mm9. Top: Screenshot from UCSC Genome Browser [77]. DNaseI hypersensitivity site (DHS) signals are from ENCODE data [8]. Bottom: Enlargement of boxed region. The 0.7 kb promoter region is depicted here. Locations of known Cast/EiJ variants [19] are depicted as green tic marks (SNPs) or blue tic marks (indels). (C) Retinal explant electroporation was used to assay the activity of the 0.7 kb *Sag* promoter region of B6 and Cast alleles. Representative images are shown here for the B6 (top) and Cast (bottom) 0.7 kb *Sag* promoter constructs driving DsRed expression. *Rho*-CBR3-eGFP served as the loading control. (D) Quantification of the *cis*-regulatory activity measured by the explant electroporation assay. Error bar represents SEM. P-value was calculated with one-tailed Wilcoxon rank-sum test.

We further focused on the *cis*-effect genes associated with retinal disease that are CBR-associated (Figure 6A). Consistent with previous observations that CBRs are enriched around retinal disease genes [6], [42], [43], we found that 38/62 (61%) were CBR-associated. Of these CBR-associated genes, 20/38 (53%) showed higher expression of the Cast/EiJ allele.

One of the CBR-associated *cis*-effect genes was *Sag*, which encodes S-arrestin, a protein important for the recovery phase of the phototransduction cascade in rods [52], [53]. Loss-of-function coding mutations in *Sag* are associated with Oguchi disease, whose clinical features include night blindness and delayed rod adaptation [54]. We found that the Cast/EiJ allele drives ∼2-fold higher *Sag* expression than the C57BL/6J allele, suggesting the presence of *cis*-regulatory variants conferring increased activity. Upon inspection of the *Sag* locus, we identified a CRX ChIP-seq peak located in the promoter/5′ UTR region and present in both CRX ChIP-seq biological replicates. This CBR corresponds to a DNaseI-hypersensitivity site (DHS) that is present at three developmental time points and is highly specific to the retina (Figure 6B) [8].

We hypothesized that *Sag* promoter variants contributed to the differential gene expression between C57BL/6J and Cast/EiJ. To test this hypothesis, we compared the activity of a 0.7 kb promoter region cloned from C57BL/6J genomic DNA (“B6 allele”) or from Cast/EiJ genomic DNA (“Cast allele”). This 0.7 kb region encompassed 5 known SNPs and 1 indel (Figure 6B). We cloned the 0.7 kb fragment upstream of a reporter gene, DsRed, and conducted a retinal explant electroporation assay to quantify CRE activity based on fluorescence (see Methods) [55].

Consistent with our hypothesis, we found that the Cast allele showed ∼22% higher CRE activity than the B6 allele (Figure 6C and 6D; P = 0.036, one-tailed Wilcoxon rank-sum test). Since *Sag* had ∼2-fold higher expression in Cast than B6, additional variants beyond this 0.7 kb promoter region likely contribute to the differential gene expression. Three other CBRs besides the promoter region were assigned to the *Sag* gene, containing 37 variants in the 1 kb windows centered on these CBRs (Supporting Information S4). Therefore, the higher expression of *Sag* in Cast compared to B6 likely results from variants in both the assayed region and other regions.

### The majority of isolated *cis* effects and isolated *trans* effects are tissue-specific

**Figure 7:**
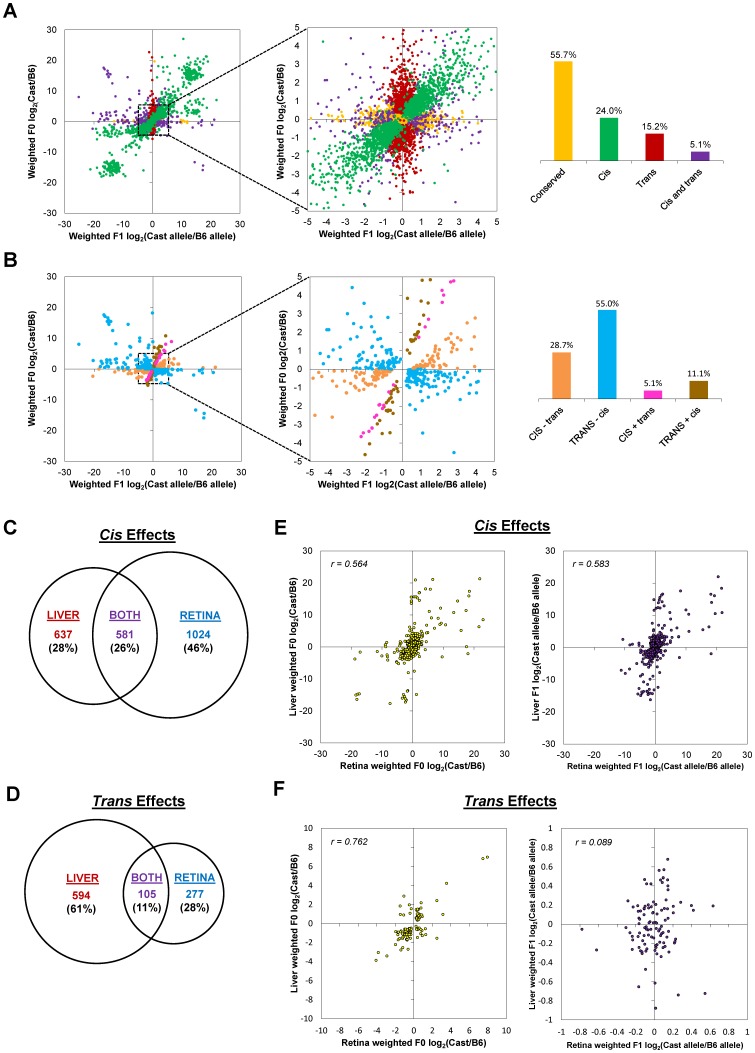
Comparison of *cis* effects and *trans* effects between liver and retina. (A) Using the same analytic pipeline as for retina, genes in the liver were classified as conserved (yellow; largely obscured), *cis* (green), *trans* (red), or *cis* and *trans* (purple). (B) *Cis*- and *trans*- regulated genes were further subcategorized as to whether the *cis* and *trans* effects acted in the same (“+” sign; pink and brown) or opposite (“−” sign; orange and blue) directions, and whether the *cis* (CAPS; orange and pink) or *trans* (CAPS; blue and brown) effect was stronger. (C) Number of genes classified as *cis* in liver and conserved in retina, *cis* in both tissues, or *cis* in retina and conserved in liver. (D) Number of genes classified as *trans* in liver and conserved in retina, *trans* in both tissues, or *trans* in retina and conserved in liver. (E) Correlation between genes classified as *cis* in both tissues. Pearson r values for F0 samples (left) and F1 samples (right) are shown. (F) Correlation between genes classified as *trans* in both tissues. Pearson r values for F0 samples (left) and F1 samples (right) are shown. Insets, magnified view.

To determine whether the isolated *cis* effects and isolated *trans* effects we identified were confined to the retina, we compared our data from retina with previously published data from liver [15] (Supporting Information S6). To ensure uniformity of analysis, we reprocessed the previously published liver data using our analytic pipeline, beginning with raw reads. After filtering 571 possibly imprinted polymorphic autosomal genes (Bayes factor >3), we were able to classify 9,865 polymorphic autosomal genes with high confidence (Figure 7A and 7B).

We found 5,494/9,865 (56%) were best modelled as conserved, 2,371/9,865 (24%) were best modelled as divergent due to *cis* effects, 1,495/9,865 (15%) as divergent due to *trans* effects, and 505/9,865 (5%) as divergent due to a combination of *cis* and *trans* effects. For genes in the latter category, 145/505 (29%) were best modelled as *CIS−trans*, 278/505 (55%) as *TRANS−cis*, 26/505 (5%) as *CIS+trans*, and 56/505 (11%) as *TRANS+cis*. Thus, as previously reported for liver, and as we found for retina, when *cis* and *trans* effects act together, they more often act to stabilize (423/505 or 84%) than to destabilize (82/505 or 16%) gene expression [15].

We then compared the classification of genes between liver and retina. To avoid misattributing tissue-specific gene expression as tissue-specific *cis* or *trans* effects, we restricted our analysis to genes classifiable in both liver and retina. In particular, for comparison of *cis*-effect genes, we required that genes be classified as *cis*-effect in one tissue and conserved in the other tissue, or *cis*-effect in both tissues. Similarly, for the comparison of *trans*-effect genes, we required that genes be classified as *trans*-effect in one tissue and conserved in the other tissue, or *trans*-effect in both tissues. Using these criteria, we found that the vast majority of *cis* effects (1,661/2,242 or 74%) were tissue-specific. Additionally, most *trans* effects (871/976 or 89%) were tissue-specific (Figure 7C and 7D; Supporting Information S7). Thus, most of the isolated *cis* and isolated *trans* effects identified were tissue-specific.

Recent studies suggest that variants in a given CRE may modulate target gene expression in a tissue-dependent manner; i.e., different tissues may show differential susceptibility to CRE variants [56]. To test for tissue-specific variant effects in our system, we examined the 581 genes classified as *cis*-effect in both liver and retina. We found a positive correlation between the expression estimates for the F0 liver and F0 retina samples (Pearson r = 0.56, two-tailed P<10^−5^), and between the expression estimates for the F1 liver and F1 retina samples (Pearson r = 0.58, two-tailed P<10^−5^) (Figure 7E). This suggests that there exists differential susceptibility between the liver and retina to CRE variants, but that there is also significant shared susceptibility.

For the 105 genes classified as *trans*-effect in both tissues, we found a positive correlation between the expression estimates for the F0 liver and F0 retina samples (Pearson r = 0.76, two-tailed P<10^−5^) (Figure 7F), suggesting that the same *trans*-acting factors regulate many of these *trans*-effect genes in both tissues. In contrast, there was no correlation between the F1 liver and F1 retina samples (Pearson r = 0.089, two-tailed P = 0.37) for these genes. This is not surprising, since by definition, *trans*-effect genes do not show AEI in F1 hybrids, and hence the log_2_ (Cast allele/B6 allele) ratios are all close to 0. Collectively, these analyses underscore the notion that *cis* effects and *trans* effects are largely tissue-specific, but when they are shared, they tend to have similar effects on gene expression.

## Discussion

Genomic techniques such as ChIP-seq and DNase-seq have greatly expanded our knowledge of *cis*-regulatory regions in various tissues and cell types in recent years [8]. Concurrently, whole-genome sequencing of thousands of individuals [57] and genome-wide association studies (GWAS) have catalogued thousands of disease-associated variants, many of which fall within regulatory regions [58]. The next phase of genomic medicine will require mapping of regulatory variants onto disease-relevant phenotypes. Here, we have taken a first step toward understanding the role of regulatory variants in retinal disease by dissecting *cis*- and *trans*-regulatory effects in the mouse retina, a tissue that models many key aspects of human retinal biology [59].

In contrast to expression quantitative trait loci (eQTL) studies, which are feasible in the human population and are largely powered to detect *cis* effects, the F1 hybrid study approach in model organisms provides tremendous power to detect both *cis* effects and *trans* effects [60]. A major finding in our study is that *cis* effects predominate in the mouse retina. While estimates of the relative contributions of *cis* effects and *trans* effects based on F1 hybrid studies in *Drosophila* and yeast vary [11]–[14], all studies acknowledge a substantial contribution of *cis* effects. The variability of estimates is likely due at least in part to methodological differences in gene expression estimates and statistical modelling. For instance, when we re-analyzed the raw data from the previously published study of *cis* and *trans* effects in mouse liver [15], we assigned a greater fraction of gene regulatory divergence to isolated *cis* and isolated *trans* effects than the original study, which assigned a greater fraction of gene regulatory divergence to combined *cis* and *trans* effects. These differences may be attributable to the fact that in our analysis pipeline, we used an updated reference transcriptome and Bayesian statistical models instead of maximum likelihood estimates (MLE).

Another key finding in our study is that the *cis* effects are largely tissue-specific, with only 26% being shared between liver and retina. Importantly, for this comparison, we included only genes with sufficient power for analysis in both tissues, and hence the observed tissue specificity is not an artifact of tissue-specific expression. Our estimate agrees well with an eQTL study of lymphoblastoid cell lines, skin, and adipose tissue in human twins, which found that 30% of *cis*-eQTLs were shared by the three tissues [61].

Predicting the effect of any given regulatory variant is a challenge, even in the face of complete genetic information, and even at the level of a molecular phenotype such as transcription factor binding [62] or, as in our case, gene expression. Moreover, regulatory variants act in combination, rather than in isolation, to modulate gene expression. Furthermore, gene expression is not always a reliable surrogate for protein levels [63], [64], and the path from protein to organismal phenotype is even more convoluted. With these layers of complexity in mind, we have taken a step toward understanding the links between *cis*-regulatory variants and retinal phenotypes by prioritizing variants within photoreceptor CREs that are associated with *cis*-effect genes.

Our work reveals that *cis*-regulatory effects predominate in the murine retina and are associated with functional *cis*-regulatory variants, with implications for retinal disease. In an approach complementary to eQTL studies, we have demonstrated a strategy for mapping *cis*-regulatory variants onto changes in gene expression by harnessing the power of inbred model organisms. Future empirical testing of such variants in living tissue, e.g., using high-throughput massively parallel reporter assays [65], [66], will further elucidate the precise causal effects of specific *cis*-regulatory variants on gene expression.

## Methods

### Ethics Statement

All experiments were conducted in strict accordance with the Guide for the Care and Use of Laboratory Animals of the National Institutes of Health (NIH), and were approved by the Washington University in St. Louis Institutional Animal Care and Use Committee (IACUC) (protocol #20110089). Animals were euthanized with CO_2_ anesthesia followed by cervical dislocation, and all efforts were made to minimize suffering.

### Animals

C57BL/6J (stock #664) and Cast/EiJ (stock #928) mice were purchased from Jackson Laboratory. Mice were maintained on a 12-hour light/dark cycle at ∼20–22°C with free access to food and water. Mating cages were maintained on 5K54 diet (LabDiet) and supplemented with autoclaved shepherd shacks (Shepherd Specialty Papers). Offspring were weaned at age 3 weeks and maintained on 5053 diet (PicoLab) until age 8 weeks, at which point they were sacrificed. Eyes were enucleated immediately after sacrifice. To minimize circadian effects [67], samples were collected at approximately the same time of day (late evening).

### Sample collection and sequencing

Each biological replicate consisted of a pool of 6–8 retinas from 8 week old male mice. Retinas were dissected in cold sterile HBSS with calcium and magnesium (Gibco) and stored at −80°C until use. Total RNA was extracted using TRIzol (Invitrogen) and purified using the RNeasy Mini Kit (Qiagen) with on-column DNaseI digestion (Qiagen). Integrity of total RNA was verified on the Agilent 2100 Bioanalyzer. Polyadenylated mRNA was captured from total RNA using Dynabeads (Invitrogen). The mRNA was fragmented and reverse-transcribed to double-stranded cDNA using random hexamers. The cDNA was blunt-ended and 3′-adenylated before ligation to sequencing adapters. Ligated fragments were amplified for 12 cycles with primers to incorporate unique sample barcodes. Libraries were subjected to paired-end 2×101 bp sequencing on the Illumina HiSeq 2000 at the Genome Technology Access Center at Washington University School of Medicine. One lane of sequencing was conducted for all F0 and F1 samples, and a second lane of sequencing was conducted for the F1 samples only.

### Read alignment and quantification

Reads were filtered and trimmed with Trim Galore! v0.2.6 [68] prior to alignment with Bowtie v0.12.9 [69] to a strain-specific reference transcriptome (for F0 data) or a hybrid reference transcriptome (for F1 data). Transcriptomes were constructed using the mouse_strain_transcriptomes.sh script within the MMSEQ package [21]. The reference transcriptomes were based on the Ensembl Release 67 cDNA files and the Wellcome Trust Mouse Genomes Project Release 2 VCF files (which use mm9/NCBI37 as the reference genome) based on November 2012 HiSeq 2×100 bp sequencing with 39x coverage of the Cast/EiJ genome [19]. MMSEQ v1.0.0 beta was used to estimate gene expression levels for the F0 samples and allele-specific gene expression levels for the F1 samples [21]. Of the 37,991 Ensembl Release 67 mouse genes, 34,964 were autosomal, of which 29,160 had known exonic polymorphisms between Cast/EiJ and C57BL/6J. Gene-level expression estimates in units roughly equivalent to FPKM (fragments per kb of transcripts per million mapped read pairs) were derived from exponentiation of the log expression estimates. For differential expression analysis of F0 samples with DESeq v1.10.1 [39], normalized count equivalents were used and a negative binomial test was performed.

### Identification of imprinted genes

Using MMDIFF, a null model (no imprinting) was compared to an imprinting model, as recently described [28]. In brief, the null model assumes that allelic expression differences are the same in F1 B6xCast and F1 CastxB6, while the imprinting model assumes that allelic expression differences have equal magnitude but opposite signs in F1 B6xCast as in F1 CastxB6. Only autosomal genes with known exonic polymorphisms between Cast/EiJ and C57BL/6J were included in this analysis.

### Mouse imprinting databases

We examined four online databases that are continually updated with known imprinted mouse genes: WAMIDEX (atlas.genetics.kcl.ac.uk) [70], MouseBook Imprinting Catalog (www.mousebook.org) [71], Geneimprint (www.geneimprint.com) [72], and Catalogue of Parent of Origin Effects (igc.otago.ac.nz) [73]. For each database, we excluded genes whose imprinting status was listed as ambiguous or disproven. To resolve nomenclature disparities between databases, we converted gene names to Mouse Genome Informatics (MGI) gene names. We combined the gene lists from the four databases into a master gene list of 189 genes, of which 143 had Ensembl Release 67 IDs and 137 were autosomal. After filtering out non-polymorphic genes, we were left with 120 autosomal Ensembl ID's, corresponding to 116 MGI genes. Each Ensembl 67 gene was then assigned a “database score” ranging from 0 to 4, indicating the number of databases that listed the gene as being imprinted (see Supporting Information S1).

### Categorization of genes according to *cis* and *trans* effects

A comparison of four models (conserved model, *cis* model, *trans* model, and *cis* and *trans* model) was performed using MMDIFF, as recently described [28]. In brief, the conserved model assumes there is no differential expression (DE) between the F0's and no allelic expression imbalance (AEI) in the F1's. The *cis* model assumes there is DE between the F0's that is equal to the AEI in the F1's. The *trans* model assumes there is DE between the F0's but no AEI in the F1's. The *cis* and *trans* model assumes that there is DE in the F0's, but it is unequal to the AEI in the F1's.

Included in the analysis were the 29,160 autosomal genes polymorphic between C57BL/6J and Cast/EiJ. In our retinal dataset, after excluding 306 possibly imprinted polymorphic autosomal genes (imprinting Bayes factor >3), we had sufficient statistical power to classify 11,484 genes confidently as conserved, *cis*, *trans*, or *cis* and *trans* based on the following criteria: the winning model must have a posterior probability >0.5, and the posterior probability of the winning model must be at least twice that of the second-best model, assuming an equal prior probability of 0.25 for each of the four models. In the previously published liver dataset [15], after excluding 571 possibly imprinted polymorphic autosomal genes (imprinting Bayes factor >3), we had sufficient statistical power to classify 9,865 genes confidently using these criteria.

Genes best modelled by a combination of *cis* and *trans* effects were then subdivided into the following categories, where x is the weighted log fold change between the strains within the F1's, and y is the weighted log fold change between the strains within the F0's [15]:

1. *CIS*−*trans* (opposite direction with *cis* stronger than *trans*): x*y>0 and |x|>|y|
2. *TRANS*−*cis* (opposite direction with *trans* stronger than *cis*): x*y<0
3. *CIS*+*trans* (same direction with *cis* stronger than *trans*): x*y>0 and |x|<|y|<|2x|
4. *TRANS*+*cis* (same direction with *trans* stronger than *cis*): x*y>0 and |y|>|2x|

### Calculation of weighted log fold change

The weighted log fold change for each gene was calculated by weighting the allele-specific posterior mean of the log expression parameter by the inverse of its posterior variance across biological replicates for each strain and subtracting the results. Let B_1_, B_2_, and B_3_ be the log expression parameters for the F0 C57BL/6J samples, and let C_1_, C_2_, and C_3_ be the log expression parameters for the F0 Cast/EiJ samples. Then the weighted log fold change between the F0 C57BL/6J samples and the F0 Cast/EiJ samples is given by 
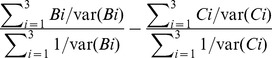
. The same approach was used to compare the two sets of F1 samples.

### Assignment of genes to CRX ChIP-seq peaks

Previously published CRX ChIP-seq data conducted on 8 week old C57BL/6 retinas [6] were used to assign wild-type (WT) CRX-bound regions (CBRs) to genes. CBRs were assigned to all autosomal and sex chromosomal Ensembl Release 67 gene transcripts using custom Perl scripts following a proximity-based algorithm as previously described: if a CBR was located within a gene, it was assigned to that gene; otherwise, it was assigned to the gene with the nearest transcriptional start site (TSS) [6].

### Batch identification of variants

Variant calls (SNPs and indels) were downloaded as Variant Call Format (VCF) files from the Wellcome Trust Sanger Institute's Mouse Genomes Project. These calls (December 2012 release) were based on the latest high-quality, high-coverage HiSeq sequencing data of the strains. The Cast/EiJ variants relative to the reference genome (C57BL/6J NCBI Build 37) were extracted at regions of interest using VCFtools v0.1.10 [74] and BEDtools v2.19.1 [75]. Only variant sites where the genotype was homozygous were included. The genomic coordinates of CBRs based on NCBI Build 37 were used. Custom Perl scripts were written to tabulate the variants for CBRs associated with Ensembl Release 67 genes.

### Identification of variants at individual regions

Individual loci of interest were manually inspected for variants by querying an online database, the Wellcome Trust Sanger Institute's Mouse Genomes Project Mouse SNP Viewer Release 1211 (NCBI Build 37), available at http://www.sanger.ac.uk/sanger/Mouse_SnpViewer/rel-1211.

### RetNet genes

Genes associated with human retinal disease in the RetNet database [51] were retrieved. Human gene symbols were converted to Mouse Genome Informatics (MGI) symbols using the MGI Batch Query [76].

### DNA constructs

Polymerase chain reaction (PCR) with Phusion High-Fidelity DNA Polymerase (New England BioLabs) was used to amplify the 0.7 kb *Sag* promoter region at −558 to +105 (mm9 chr1:89,699,697–89,700,359) relative to the TSS. Genomic DNA purified from C57BL/6J and Cast/EiJ liver tissue was used as the template for the B6 and Cast construct, respectively. The forward primer 5′-TGAGGCAATGACACTTGGTC-3′ and reverse primer 5′-GCAGGGAGCTGATTGGATTA-3′ with *Xho*I and *Eco*RI restriction enzyme site overhangs, respectively, were used. The fragments were subcloned upstream of DsRed in the no-basal vector (described previously in [4]) using the *Sal*I (compatible with *Xho*I) and *Eco*RI sites. Constructs were confirmed with Sanger sequencing that encompassed the entire 0.7 kb region. We note that based on our high-quality Sanger sequencing of this region, the genomic DNA of our Cast/EiJ mice differed from the reference Cast/EiJ sequence [19] by two bases at chr1:89,700,191 (A→C) and chr1:89,700,187 (A→C), as confirmed by Sanger sequencing three different Cast/EiJ mice (representing the three Cast/EiJ RNA-seq biological replicates)

### Retinal explant electroporation and quantification of promoter activity

Electroporation and explant culture of mouse retinas were performed as described previously [55]. In brief, retinas were dissected from newborn (P0) CD-1 mouse pups and coelectroporated with one of the *Sag* promoter DsRed constructs and a control green fluorescent protein (GFP) reporter that expresses in rod photoreceptors, *Rho*-CBR3-eGFP [6], each at a concentration of 0.5 µg/µL. Retinas were grown in explant culture and harvested 8 days later, whereupon they were fixed and whole-mounted for quantitative imaging of DsRed fluorescence intensity normalized to GFP fluorescence intensity using a monochromatic camera (Hamamatsu ORCA-AG), as described [55]. For each *Sag* promoter construct, 10–11 retinas were quantified. Representative images using a color camera (Olympus DP70) were also taken (see Figure 6C). For all retinal imaging, 4X magnification was used, and the exposure times for the red and green channels were consistent across retinas.

### Data Access

RNA-seq, MMSEQ, and MMDIFF data have been deposited in Gene Expression Omnibus (GEO, http://www.ncbi.nlm.nih.gov/geo/) (accession number GSE60545).

## Supporting Information

Supporting Information S1 **Analysis of parent-of-origin effects in the retina.** This Excel file summarizes the analysis of all 29,160 polymorphic autosomal genes (ranked by Bayes factor) for parent-of-origin effects, as determined by allele-specific expression estimates in the reciprocal F1 hybrids using MMSEQ and MMDIFF. Red shading, log_e_ expression measures for maternally derived alleles. Blue shading, log_e_ expression measures for paternally derived alleles. Yellow shading, weighted log_2_ fold change values.
(XLSX)

Supporting Information S2 **RNA-seq differential expression analysis of F0 C57BL/6J and F0 Cast/EiJ adult retinas.** This Excel file summarizes the DESeq analysis of F0 samples. The first sheet contains all 37,991 genes, the second sheet contains all 34,964 autosomal genes, and third sheet contains the 3,799 autosomal genes found to be differentially expressed at 5% false discovery rate (FDR). Each sheet is sorted on FDR-adjusted P-values. Unique CBR identifiers and tally of CBR read counts are based on data from [6].
(XLSX)

Supporting Information S3 **Classification of gene regulatory divergence in the retina.** This Excel file summarizes the classification of *cis* and *trans* effects in the F0 and F1 retinal samples based on MMDIFF. The first sheet contains the primary classification (conserved, *cis*, *trans*, or *cis* and *trans*) of the 11,484 classifiable genes. The second sheet contains the subcategorization of the 717 genes whose regulatory divergence is due to a combination of *cis* and *trans* effects. Orange shading, posterior probability of model based on prior probability of 0.25. Yellow shading, weighted log_2_ fold change values. Unique CBR identifiers and tally of CBR read counts are based on data from [6].
(XLSX)

Supporting Information S4 **Analysis of Cast/EiJ variants within CBRs.** This Excel file summarizes the analysis of Cast/EiJ variants (SNPs and indels) within CBRs. The first sheet contains a list of information for all 10,212 CBRs, including the locations of variants within the central 1 kb of each CBR [6]. “Doublehit” CBRs are those found in both CRX ChIP-seq replicates. “Singlehit” CBRs are those found in one CRX ChIP-seq replicate. The second sheet contains a list of all 37,991 Ensembl 67 genes, the unique CBR identifiers assigned to each gene, and the number of variants within the central 1 kb of all CBRs associated with each gene. The third sheet gives the number of variants within the central 1 kb of CBRs associated with *cis*-effect genes in the retina. The fourth sheet gives the number of variants within the central 1 kb of CBRs associated with *trans*-effect genes in the retina.
(XLSX)

Supporting Information S5 ***Cis*** **effect genes associated with retinal disease.** This Excel file lists the 62 genes that were classified as *cis*-effect in the retina and whose human orthologues were found in the RetNet [51] database of human retinal disease genes. Yellow shading, weighted log_2_ fold change values. Unique CBR identifiers and tally of CBR read counts are based on data from [6]. Note that the unique CBR identifiers can be cross-referenced with the first sheet in Supporting Information S4, which lists the locations of the CBRs by CBR identifier. Expression level values (in FPKM units) are provided. Light gray shading, F0 C57BL/6J values. Light brown shading, F0 Cast/EiJ values. Dark gray shading, F1 B6 allele values. Dark brown shading, F1 Cast allele values.
(XLSX)

Supporting Information S6 **Classification of gene regulatory divergence in the liver.** This Excel file summarizes the re-analysis of previously published F0 and F1 liver data [15]. Note that F1i is equivalent to F1 B6xCast (resulting from B6 male×Cast female) and F1r is the reciprocal cross. The first sheet contains the 9,865 classifiable genes after filtering non-polymorphic genes and genes with imprinting Bayes factor >3. The second sheet contains the subcategorization of the 505 genes whose regulatory divergence is due to a combination of *cis* and *trans* effects. Orange shading, posterior probability of model based on prior probability of 0.25. Yellow shading, weighted log_2_ fold change values.
(XLSX)

Supporting Information S7 **Comparison of** ***cis*** **and** ***trans*** **effects in the liver and retina.** This Excel file summarizes the comparison of *cis*-effect genes and *trans*-effect genes in the liver and retina. The first sheet contains the Ensembl ID's of *cis* effects and *trans* effects specific to, or shared between, the liver and retina. The second sheet contains the F0 and F1 weighted log_2_ fold change values for genes classified as *cis*-effect in both tissues. The third sheet contains the F0 and F1 weighted log_2_ fold change values for genes classified as *trans*-effect in both tissues.
(XLSX)
